# Bidirectional Causal Associations Between Same-Sex Attraction and Psychological Distress: Testing Moderation and Mediation Effects

**DOI:** 10.1007/s10519-022-10130-x

**Published:** 2022-12-15

**Authors:** Olakunle A. Oginni, Kai X. Lim, Qazi Rahman, Patrick Jern, Thalia C. Eley, Frühling V. Rijsdijk

**Affiliations:** 1 The Social, Genetic and Developmental Psychiatry Centre, Institute of Psychiatry, Psychology and Neuroscience, King’s College London, Denmark Hill, SE5 8AF UK; 2 Department of Mental Health, Obafemi Awolowo University, Ile-Ife, Nigeria; 3 Department of Psychology, Institute of Psychiatry, Psychology and Neuroscience, King’s College London, London, UK; 4 Department of Psychology, Åbo Akademi University, Åbo, Finland; 5 Department of Psychology, Faculty of Social Sciences, Anton de Kom University, Paramaribo, Suriname

**Keywords:** Same-sex attraction, Psychological distress, Minority stress, Causality, Mediation, Moderation

## Abstract

Only one study has examined bidirectional causality between sexual minority status (having same-sex attraction) and psychological distress. We combined twin and genomic data from 8700 to 9700 participants in the UK Twins Early Development Study cohort at ≈21 years to replicate and extend these bidirectional causal effects using separate unidirectional Mendelian Randomization-Direction of Causation models. We further modified these models to separately investigate sex differences, moderation by childhood factors (retrospectively-assessed early-life adversity and prospectively-assessed childhood gender nonconformity), and mediation by victimization. All analyses were carried out in OpenMx in R. Same-sex attraction causally influenced psychological distress with significant reverse causation (beta = 0.19 and 0.17; 95% CIs = 0.09, 0.29 and 0.08, 0.25 respectively) and no significant sex differences. The same-sex attraction → psychological distress causal path was partly mediated by victimization (12.5%) while the reverse causal path was attenuated by higher childhood gender nonconformity (moderation coefficient = −0.09, 95% CI: −0.13, −0.04).

**Supplementary Information:**

The online version contains supplementary material available at 10.1007/s10519-022-10130-x.

## Introduction

**Fig. 1:**
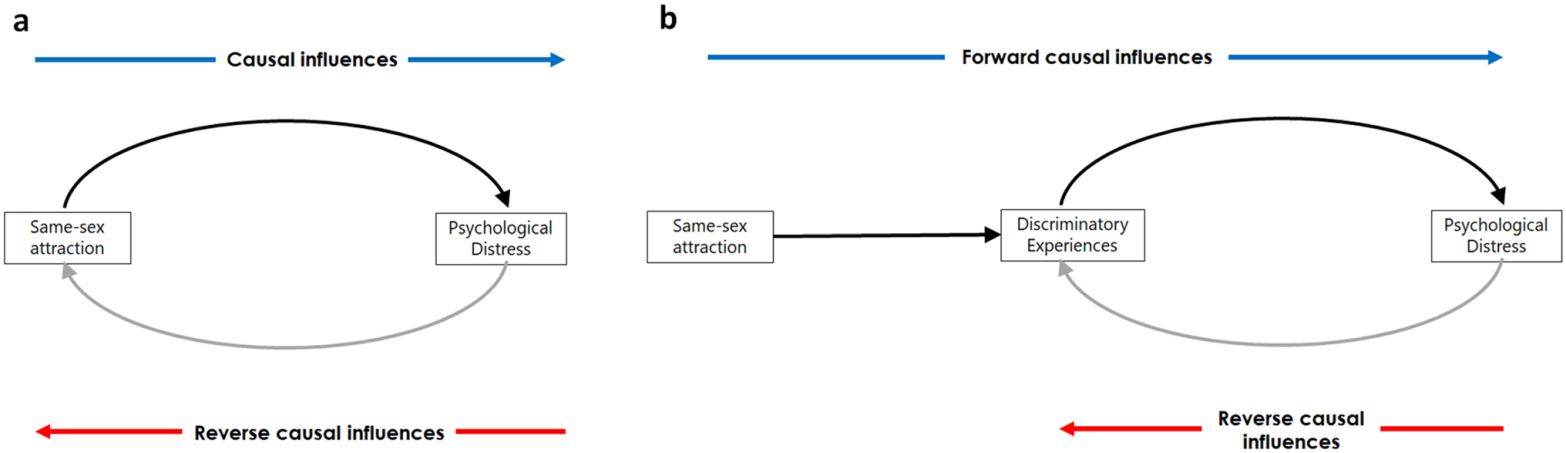
Schematic illustration of the bidirectional relationships between same-sex attraction and psychological distress (**a**) and proposed mediation of these relationships by discriminatory experiences (**b**); a component of minority stress (Meyer, 2003). *Note:* **a** depicts bidirectional (i.e., forward and reverse) causal relationships between same-sex attraction (as an index of sexuality-related stress; Schwartz and Meyer, 2010) and increased psychological distress but without a specific mechanism (e.g., as demonstrated by Oginni et al., 2022a). This relationship may be further decomposed into (i) a mediation of the ‘forward’ causal effect of same-sex attraction on psychological distress by discriminatory experiences, and (ii) a reciprocal causal relationship between discriminatory experiences and psychological distress (**b**)

Psychological distress (depressive and anxiety symptoms) is significantly higher among individuals who are sexually attracted to people of the same sex (including those who identify as being lesbian, gay or bisexual) compared to those who are exclusively heterosexual (King et al., 2008; Plöderl & Tremblay, 2015; Semlyen et al., 2016). Minority stress theory attributes these mental health disparities to the stressful consequences of having a sexual minority identity (Meyer, 2003) which, in turn, is based on same-sex sexual attraction (Bailey et al., 2016). Specific minority stress factors include experiences of discrimination or victimization, concealment of sexual orientation and internalized sexuality-related stigma (Meyer, 2003). Related processes include rejection sensitivity (Feinstein, 2020), emotional dysregulation (Hatzenbuehler, 2009) and excessive rumination (Timmins et al., 2020). Some of these minority stressors (e.g., sexuality-related discrimination and internalized stigma, and concealment) are predominantly experienced by same-sex attracted individuals. Apart from these, sexual minority individuals may further experience intangible sexuality-related stresses, which also contribute to the observed health disparities but are difficult to measure (Schwartz & Meyer, 2010). Hence, consistent with previous theory and research (Oginni et al., 2022a; Schwartz et al., 2010), in the present study, sexual minority status (e.g., having same-sex sexual attraction) is regarded as an index of measurable and unmeasurable sexuality-related stress (Fig. 1).

However, the links between minority stress and mental health disparities among same-sex attracted individuals is mostly cross-sectional, which limits causal inference (Bailey, 2020). Thus, while there is a robust cross-sectional evidence base (Dürrbaum & Sattler, 2020), this is not sufficient to inform intervention design. Furthermore, the few studies that utilize a longitudinal design only test for prospective associations rather than the direction of causality (e.g., Bränström, 2017). In contrast, studies that have investigated causality tend to utilize samples comprising only sexual minority participants (Rosario et al., 2002). Although this within-group analysis allows the investigation of the effects of minority stressors which are unique to same-sex attracted individuals, the absence of a comparison heterosexual group limits the extent to which the findings are indicative of health disparities between same-sex attracted and heterosexual individuals (Meyer, 2010; Schwartz & Meyer, 2010). A superior approach would be to investigate stressors in both same-sex attracted and heterosexual samples and determine the extents to which they account for observed health disparities.

Recently, an alternative approach was used to provide evidence for bidirectional causal relationships between sexual minority stress (indexed by sexual minority status) and psychological distress (Oginni et al., 2022a). Specifically, the authors used a model that combines the Direction of Causation twin model with Mendelian Randomisation using polygenic scores as instruments to determine causality—the Mendelian Randomisation-Direction of Causation (MRDoC) model (Minică et al., 2018). The advantage of the MRDoC model is that it enables the relaxation of certain assumptions of each individual method (Minică et al., 2018), which in turn leads to less-biased estimates of causal path coefficients (Minică et al., 2020). More details of this analytic approach are provided in the Methods section.

Previous research indicates that the disparity in depressive and anxiety disorders among sexual minority compared to heterosexual individuals is greater among males compared to females (King et al., 2008). Furthermore, there is evidence that early-life adversities and childhood gender nonconformity moderate the cross-sectional phenotypic and genetic and environmental correlations between sexual minority status and depressive and/or anxiety symptoms (D’Augelli et al., 2006; Oginni et al., 2022b). Finally, in explaining their finding of bidirectional causal effects (Oginni et al., 2022a), the authors speculated that the reverse causal effect indicated a positive feedback effect of psychological distress on sexual minority stress processes. However, this was not tested empirically, for example, by specifically testing mediation of the causal effects by measured minority stress factors (Fig. 1).

The objectives of the present study were therefore to: (i) use the MRDoC model to replicate the bidirectional causal associations between same-sex attraction (indexing sexuality-related distress) and psychological distress in a different twin sample; (ii) test sex differences in the proposed bidirectional causal relationships; (iii) test moderation of the causal paths by early-life adversities and childhood gender nonconformity; and (iv) test whether victimization (a proxy for minority stress) mediates these causal relationships.

We hypothesized that there would be bidirectional causal paths between same-sex attraction and psychological distress (i.e., stress associated with sexual minority status will cause psychological distress which can in turn potentiate the former), and that these effects would be stronger in men compared to women. Based on existing evidence (D’Augelli et al., 2006; Oginni et al., 2022b), we further hypothesized that the bidirectional causal links between same-sex attraction and psychological distress would be stronger at higher levels of early-life adversity and childhood gender nonconformity. Finally, we hypothesized that the causal path from same-sex attraction to psychological distress would be mediated by victimization but we were agnostic about mediation of the reverse causal path.

## Methods

### Sample

The sample comprised twin participants in the 21-year wave of the UK Twins Early Development Study (TEDS) cohort. Data were collected in two phases between June 2017 and February 2019. About 16,810 participating families were originally contacted; of these, both email and paper invitations were sent to 10,571 and 8611 families who respectively indicated willingness to participate in the first and second phases of data collection. In both phases, data were collected using either a mailed paper booklet, a mobile phone application, or a web-based platform as preferred by the participants. The final sample comprised 9697 and 8718 individuals (response rates of 45.9% and 50.6% respectively) from the first and second phases of data collection respectively, and these rates are comparable those from mailed surveys (Guo et al., 2016; Oginni et al., 2020). Further details of the recruitment and response rates are available from the TEDS website (https://www.teds.ac.uk/datadictionary/studies/21yr.htm) and from previous descriptions (Haworth et al., 2013; Rimfeld et al., 2019). Zygosity was assessed using a parent-reported measure of physical similarity during childhood; which, when compared to DNA testing, correctly identified 95% of twins (94% of monozygotic and 98% of dizygotic twins; Price et al., 2000).

Up to 285 individuals were excluded due to unknown zygosity and missing essential background information. The number of participants with sufficient responses per variable ranged from 7915 to 9104 (Supplementary Table S1) corresponding to 91–96% of those who responded during phases 1 and 2 of data collection respectively. Ethical approval was granted by King’s College London’s ethics committee for the Institute of Psychiatry, Psychology and Neuroscience and informed consent was obtained from all participants.

### Measures in main analyses

Same-sex attraction was used as an index for sexuality-related stress (Oginni et al., 2022a; Schwartz & Meyer, 2010). It was assessed using a single question about the sex of people to whom participants are sexually attracted, with responses including ‘Always male’, ‘Mostly male but sometimes female’, ‘Equally males and females’, ‘Mostly female but sometimes male’, and ‘Always female’. These were differentially recoded one to five to correspond to ‘Always opposite sex’, ‘Mostly opposite sex but sometimes same sex’, ‘Equally same and opposite sexes’, ‘Mostly same sex but sometimes opposite sex’ and ‘Always same sex’ in male and female participants. Thus, higher scores indicated greater same-sex attraction. Participants who selected either of two further responses: ‘Little or no sexual attraction’ and ‘Unsure or do not know’ were excluded from the analyses (*n* = 216) because the small sample size of this subgroup precludes meaningful comparisons. Same-sex attraction was specified as a liability threshold variable with four thresholds in which the observed categories are assumed to reflect imperfect thresholds of a latent normally-distributed liability to same-sex attraction (Kline, 2015; Rijsdijk & Sham, 2002).

Depressive symptoms were assessed using a shortened 8-item version of the Mood and Feelings Questionnaire (Angold et al., 1995). Each item rates the presence and severity of depressive symptoms over the prior two weeks via a three-point Likert scale ranging from 0 (Not at all) through 1 (Somewhat true) to 2 (True). Total scores were derived by summing the item responses whereby higher scores indicated more severe depressive symptoms. Cronbach’s alpha in the present study was 0.87 and continuous scores were used in analyses. Only participants who responded to at least 6 items were included in analyses.

Symptoms of Generalized Anxiety Disorder (GAD) were assessed using the 10-item Severity Measure for Generalized Anxiety Disorder—Adult (Craske et al., 2013). The items rate the frequency of each symptom of GAD over the preceding week using a five-point Likert scale ranging from 0 (Never) to 4 (All of the time). The responses were summed to derive a total score with higher scores indicating more severe GAD symptoms. The Cronbach’s alpha was 0.91 and total scores were used in analyses. Only participants who responded to at least seven items were included in analyses.

Victimization was assessed using the 16-item victimization subscale of the Multidimensional peer-victimization scale (Mynard & Joseph, 2000). The items rate the frequency of physical, social, verbal and cyber bullying on a three-point Likert scale with responses including ‘Not at all’, ‘Once’, and ‘More than once’ coded 0, 1 and 2 respectively. The responses were summed with higher scores indicating higher victimization. The Cronbach’s alpha was 0.87 and continuous scores were used in analyses. Only participants who responded to at least 12 items were included in analyses.

Early-life adverse experiences were retrospectively assessed using eight questions about physical and psychological abuse derived from items in the Avon Longitudinal Study of Parents and Children (https://www.teds.ac.uk/datadictionary/studies/measures/21yr_ measures.htm). Each item was scored on a 5-point Likert scale ranging from 0 (Never) to 4 (Very often). Total scores were derived by summing the responses with higher scores indicating greater early-life adverse experiences. The Cronbach’s alpha was 0.86 and continuous scores were used in analyses. Only participants who responded to at least 6 items were included in analyses.

Childhood gender nonconformity was prospectively assessed using the 24-item Pre-School Activities Inventory which was completed by the mothers when the twins were aged four years (Golombok & Rust, 1993). The questionnaire assesses stereotypical gender-specific play, activities and other characteristics. Responses are scored on a five-point Likert scale ranging from 1 (never) to 5 (very often). These were summed and transformed so that higher scores indicated higher male-typical behaviors. These scores were standardized, and scores for male participants multiplied by −1 so that higher scores in male and female participants were indicative of higher childhood gender nonconformity (Oginni et al., 2019). The Cronbach’s alpha for this questionnaire in the present study was 0.65 and continuous scores were used in analyses. Only participants who responded to at least 18 items were included in analyses.

For participants included in the analyses (those with item-level missingness ranging between 25% and 30% as described above), missing responses were prorated; furthermore, maximum likelihood estimation was used for structural equation modelling which is robust to missing data (Allison, 2003).

### Covariates

Participants’ age and birth sex were ascertained using single questions asked during earlier waves of data collection.

### Polygenic scores

Genotype data were collected at ages 12- and 16-years using cheek swab and saliva samples respectively. With these, polygenic scores were calculated for same-sex attraction,^1^ depressive and anxiety symptoms as the weighted sum of the number of genome-wide trait-associated alleles weighted by effect sizes using the Bayesian-based LDpred (Vilhjálmsson et al., 2015). Effect sizes were derived from genome-wide association studies for same-sex behavior (Ganna et al., 2019), and depressive and anxiety symptoms (Howard et al., 2019; Purves et al., 2020). Further details of genomic data processing and polygenic score calculation are described in the supplementary material.

### Latent factors

Four latent factors were specified to facilitate analyses as follows: Same-sex attraction (with the corresponding variable as its indicator), psychological distress (with depressive and anxiety symptoms as indicators), genetic propensity for same-sex attraction (with same-sex attraction polygenic scores as the indicator), and genetic propensity for psychological distress (with polygenic scores for depressive and anxiety symptoms as indicators).

### Statistical analyses

#### Data preparation and summary statistics

Data cleaning and preliminary descriptive analyses were carried out using SPSS version 26 and STATA version SE 14.2. Subsequent analyses were carried out using OpenMx (Neale et al., 2016). The study variables were residualized to control for the covariates as is standard practice in twin modelling to reduce the potential inflation of the latent shared environmental effects (McGue & Bouchard, 1984). The residuals were further normalized to conform to parametric assumptions necessary for subsequent analyses.

#### Phenotypic correlations

Phenotypic factor correlations were estimated using maximum likelihood estimation and constraints applied to facilitate estimation of many correlations using a reduced set of statistics. Specifically, we constrained within-person correlations to be equal across birth order and zygosity; within-trait and cross-trait correlations were allowed to vary by zygosity while the latter were constrained to be symmetrical.

#### Multivariate genetic model fitting

Next, we used a Cholesky decomposition to parse the variances and covariance of the same-sex attraction and psychological distress latent factors, and the residual variances of their indicator variables into latent additive genetic (A) and shared (C) and individual-specific (E) environmental components. *A* influences index the sum of the effects of multiple genetic loci across the genome, *C* influences encompass environmental influences which make family members similar to one another and *E* influences include environmental factors which make family members different from one another including measurement error (Neale & Cardon, 2013; Rijsdijk & Sham, 2002). To estimate these components, the classical twin design assumes that monozygotic and dizygotic twin pairs raised together are 100% and 50% genetically similar respectively but are influenced by their shared environment to the same extent (Rijsdijk & Sham, 2002).

#### Mendelian randomization-direction of causation (MRDoC) models

We specified two separate MRDoC models to test whether same-sex attraction causally influenced psychological distress (MRDoC Model 1) and whether reverse causal influences also flowed from psychological distress to same-sex attraction (MRDoC Model 2). The MRDoC model combines Mendelian Randomization (MR) with the Direction of Causation (DoC) twin model which both use cross-sectional data to determine the direction of causation under specific conditions (Minică et al., 2018). The Direction of Causation model requires different patterns of variance component influences for each variable to test bidirectional causal effects (Heath et al., 1993). In contrast, Mendelian Randomization tests a unidirectional causal relationship by specifying individual genetic variants as instruments (Burgess & Thompson, 2011). To be valid, an instrument must be associated with the proposed exposure variable, independent of confounders and not independently associated with the outcome variable (Burgess & Thompson, 2011). However, most variables do not have sufficiently distinct ACE influences (Polderman et al., 2015), and single genetic variants are prone to weak instrument bias (Burgess & Thompson, 2011) (Fig. 1).

**Fig. 2:**
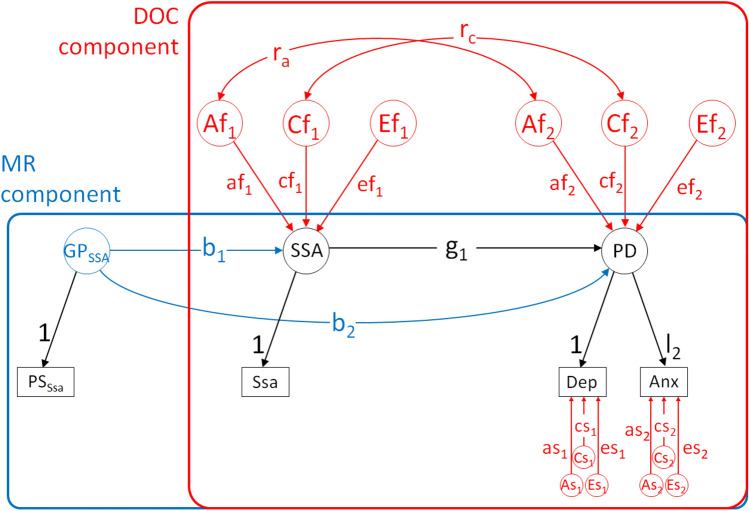
Mendelian randomization-direction of causation (MRDoC) model. *Note*. The MRDoC model combines the twin Direction of Causation (DOC) model (in the red box) with Mendelian Randomization (MR, in the blue box) to determine causal effects. In MRDoC Model 1, Same-Sex Attraction (SSA) is specified as the predictor and Psychological Distress (PD—indicated by Depressive and Anxiety symptoms (Dep and Anx respectively)) as the outcome. Genetic Propensity for Same-Sex Attraction (GP_SSA_, indicated by polygenic scores for same-sex attraction—PS_Ssa_) is specified as the outcome. b_1_ and b_2_ represent the instrumental and pleiotropic paths respectively and g_1_ represents the causal path. Af_1–2_, Cf_1–2_ and Ef_1–2_ = Additive genetic and shared and individual-specific environmental influences on SSA and PD respectively; af_1–2_, cf_1–2_ and ef_1–2_ = their path coefficients; As_1–2_, Cs_1–2_ and Es_1–2_ = Residual additive genetic and shared and individual-specific environmental influences on Depressive (Dep) and Anxiety symptoms (Anx) respectively; and as_1–2_, cs_1–2_ and es_1–2_ = their path coefficients. In MRDoC model 2, Genetic propensity for Psychological Distress (GP_PD_), PD and SSA were specified as the instrument, predictor and outcome respectively

In combining both models, the MRDoC model tests unidirectional causation, does not require differential variance components, assumes no *E* correlations between variables of interest and overcomes weak-instrument bias by specifying polygenic scores as an instrument (Minică et al., 2018). The MRDoC model adjusts for potential violation of the properties of a valid instrument by specifying a pleiotropic path between the polygenic score and the outcome variable (Fig. 2). Covariance paths between *A* and *C* components further adjust for residual confounding which further reduces bias in the estimated causal path coefficient.

To assess sex differences in the causal paths, we compared heterogeneity MRDoC models (for MRDoC models 1 and 2) in which all the parameters were allowed to differ by sex with corresponding ones in which the causal paths were constrained to be equal in males and females (homogeneity models). Model comparison was done using likelihood-ratio testing. Preliminarily, we tested for sex differences in the distributions of the variables (using linear regressions with each variable specified as a separate outcome, sex as the predictor while controlling for familial clustering using the cluster option in STATA; Krebs et al., 2020) and their phenotypic correlations.

To test moderation by early-life adversities and childhood gender nonconformity, we specified moderation coefficients on the causal paths in MRDoC models 1 and 2 (Figure S2, Supplementary material). To minimize the false positive moderation of causal effects, we also specified moderation coefficients for the instrumental and pleiotropic paths, the *A* and *E* variance component paths and the *A* covariance path (Purcell, 2002). Furthermore, as cross-paths for the cross-twin within-pair correlations for the moderators were not specified in the moderation models, we used the extended fixed regression approach which controls for the main effects of the co-twin’s moderator variable (early-life adversity or childhood gender nonconformity) on each twin’s variables of interest: same-sex attraction and psychological distress (Van der Sluis et al., 2012).

#### Exploratory analyses

We further tested whether the causal paths in MRDoC Models 1 and 2 were mediated by victimization (Supplementary Figure S1). These analyses tested whether the causal influences of same-sex attraction on psychological distress were partly explained by victimization (MRDoC Model 3) and whether the reciprocal causal path between psychological distress and same-sex attraction was partly explained by victimization (MRDoC Model 4). Similar to the unmediated MRDoC model (Minică et al., 2018), the covariances between E influences on same-sex attraction, victimization and psychological distress were fixed to 0.

### Preregistration

This study was pre-registered at https://osf.io/dgcm6/ and we departed from the proposed analyses as follows: Risky sexual behavior (RSB) was excluded from analyses because there was insufficient power to determine the direction of causation using the MRDoC model (i.e., both bidirectional causal path coefficients were similar in magnitude to the phenotypic correlation coefficient), possibly because of its small correlation with sexual orientation. Furthermore, we tested mediation of the MRDoC models directly by specifying victimization as a mediator in the model rather than indirectly by residualizing same-sex attraction and psychological distress on victimization before modelling.

## Results

### Descriptive statistics

The mean age of the participants was 22.3 (± 0.92) years. About 81% of the participants reported being always attracted to the opposite sex while the remainder reported varying degrees of same-sex attraction which ranged from 1.9% being mostly same-sex attracted and 11.5% being mostly attracted to the opposite sex (Table 1). More females (21.4%) reported being same-sex attracted compared to male participants (15.3%; F[1] = 29.43; *p* < 0.001). The median scores for depressive and anxiety symptoms, victimisation and early-life adversity were 3.0, 5.0, 2.0 and 4.0 respectively (Inter-quartile ranges: 6.0, 8.0, 5.0 and 5.0 respectively). The depressive and anxiety symptoms scores were higher in female participants (*p* < 0.001 in both instances) while the median victimisation score was significantly higher in male participants (*p* < 0.001 respectively). However, early-life adversity and childhood gender nonconformity did not significantly differ by sex.

**Table 1:** Descriptive statistics of observed variables and sex comparisons

Variable	Total *n* (%)^a^/Median (IQR)^b^/Mean (SD)^c^	Male *n* (%)^a^/Median (IQR)^b^/Mean (SD)	Female *n* (%)^a^/Median (IQR)^b^/Mean (SD)	F[df]/(*p*-value)
Same-sex attraction^a^				29.43[1]/(< 0.001)^d^
Always opposite sex	7309 (80.90)	2889 (84.75)	4420 (78.56)	
Mostly opposite sex but sometimes same sex	1042 (11.53)	213 (6.25)	829 (14.74)	
Equally same and opposite sexes	182 (2.01)	34 (1.00)	148 (2.63)	
Mostly same sex but sometimes opposite sex	175 (1.94)	62 (1.82)	113 (2.01)	
Always same sex	327 (3.62)	211 (6.19)	116 (2.06)	
Depressive symptoms^b^	3.0 (6.00)	3.0 (5.00)	4.0 (6.50)	168.50[5384]/(< 0.001)
Anxiety symptoms^b^	5.0 (8.00)	4.0 (7.00)	6.0 (8.00)	193.20[4962](< 0.001)
Victimization^b^	2.0 (5.00)	2.0 (6.00)	1.0 (5.00)	44.43[4946]/(< 0.001)
Early-life adversity^b^	4.0 (5.00)	4.0 (5.00)	4.0 (5.00)	0.97[5032]/(0.32)
CGN^c^	−0.01 (1.00)	−0.01 (1.00)	−0.01 (0.99)	0.00[7823]/(1.00)

*df* degrees of freedom, *CGN* Childhood gender nonconformity

^a^*n* (%)

^b^Median (IQR)

^c^Mean (SD)

^d^Ordinal logistic regression

### Phenotypic factor correlations

There was a significant positive correlation between same-sex attraction and psychological distress (*r* = 0.30; 95% CI: 0.27, 0.33) such that increasing same-sex attraction was associated with higher psychological distress (Table 2). Genetic propensity for same-sex attraction (GP_SSA_) was significantly associated with same-sex attraction (*r* = 0.08; 95% CIs = 0.04, 0.12) but not psychological distress (*r* = 0.01; 95% CIs = −0.02, 0.05). Genetic propensity for psychological distress (GP_PD_) was also associated with psychological distress (*r* = 0.17, 95% CI: 0.13, 0.21) and same-sex attraction (*r* = 0.08, 95% CI: 0.03, 0.13). The significant association between GP_PD_ and same-sex attraction (SSA) was consistent with pleiotropy of GP_PD_. Victimisation was also significantly correlated with same-sex attraction and psychological distress (*r* = 0.16 and 0.41; 95% CIs = 0.13, 0.19; and 0.39, 0.43 respectively). These phenotypic factor correlations were consistent with the variable correlations and are summarized in Table S2 in the Supplementary Material.

**Table 2:** Phenotypic correlations between latent factors standardized factor loadings and residual variance paths

	Same-sex attraction (1)	Psychological distress (2)	Victimization (3)	GP_SSA_ (4)	GP_PD_ (5)
Within-person					
1	1.00				
2	.30 (.27, .33)	1.00			
3	.16 (.13, .19)	.41 (.39, .43)	1.00		
4	.08 (.04, .12)	.01 (−.02, .05)	−.01 (−.04, .02)	1.00	
5	.08 (.03, .13)	.17 (.13, .21)	.10 (.06, .14)	.16 (.13, .19)	1.00
Cross-twin					
Monozygotic					
1	.56 (.49, .63)				
2	.20 (.15, .25)	.55 (.50, .60)			
3	.12 (.07, .17)	.22 (.18, .26)	.34 (.29, .38)		
4	.08 (.04, .12)	.01 (−.02, .05)	−.01 (−.04, .02)	1.00	
5	.08 (.03, .13)	.17 (.13, .21)	.10 (.06, .14)	.16 (.13, .19)	1.00
Dizygotic					
1	.22 (.15, .30)				
2	.13 (.09, .18)	.24 (.19, .29)			
3	.05 (.01, .09)	.11 (.08, .15)	.14 (.10, .19)		
4	.02 (−.03, .06)	.00 (−.04, .03)	−.02 (−.06, .01)	.50	
5	.02 (−.04, .08)	.07 (.02, .12)	.04 (.00, .09)	.05 (.02, .08)	.50
Variables	Standardized factor loadings	Standardized error variance path			
Same-sex attraction	1.00	.00			
Dep symptoms	.62 (.60, .63)	.78 (.77, .80)			
Anx symptoms	.87 (.86, .87)	.50 (.49, .51)			
Victimization	1.00	.00			
PS_SSA_	1.00	.00			
PS_Dep_	.68 (.66, .69)	.73 (.72, .75)			
PS_Anx_	.65 (.64, 67)	.76 (.75, .77)			

The factor loadings for factors with single indicator variables (Same-sex attraction, Victimization and GP_SSA_) were fixed to 1 while the residual variances were fixed to 0 for identification

*GP*_*SSA*_*, GP*_*PD*_ Genetic Propensities for Same-Sex Attraction and Psychological Distress respectively, *Dep* and *Anx* symptoms Depressive and Anxiety symptoms respectively, *PS*_*SSA*_*, PS*_*Dep*_ and *PS*_*Anx*_ Polygenic Scores for same-sex attraction, depressive symptoms and anxiety symptoms

The cross-twin correlations for each factor among monozygotic twins were more than twice those in dizygotic twins indicating genetic rather than shared environmental influences on the factor variances (Table 2).

### Biometric genetic models

#### Preliminary cholesky decomposition

The heritability estimates for same-sex attraction, psychological distress and victimization in the full *ACE* model were 54%, 54% and 32% (95% CIs: 0.40, 0.60; 0.43, 0.60 and 0.23, 0.36 respectively [Supplementary Table S3]). The standardized *E* contributions to the variances of the three factors were 46%, 46% and 68% (95% CIs: 0.40, 0.53; 0.40, 0.51 and 0.64, 0.72 respectively). As *C* influences on all three factors were each 0% and did not lead to a significant worsening of fit when dropped from the model (χ^2^[7] = 0.00, *p* = 1), this component was dropped from subsequent analyses and we report estimates from the *AE* model (Table 3).

**Table 3:** Standardized additive genetic and individual-specific environmental influences on the variances and covariances between the latent factors

h^2^	Same-sex attraction (1)	Psychological Distress (2)	Victimization (3)
1	.54 (.47, .60)		
2	.71 (.57, .85)	.49 (.44, .54)	
3	.74 (.49, .95)	.55 (.47, .63)	.32 (.28, .36)
e^2^			
1	.46 (.40, .53)		
2	.29 (.15, .43)	.51 (.46, .56)	
3	.26 (.05, .51)	.45 (.37, .53)	.68 (.64, .72)
Variables	Factor loading	As	Es
Same-sex attraction	1	–	–
Depressive symptoms	.62 (.60, .63)	.10 (.07, .13)	.52 (.49, .55)
Anxiety symptoms	.87 (.86, .87)	.04 (.03, .05)	.21 (.20, .22)
Victimization	1	–	–

h^2^ and e^2^ = standardized additive genetic, and individual-specific environmental influences on factor variances (the diagonals) and covariances (the off-diagonals)

#### Causal associations between same-sex attraction and psychological distress

**Fig. 3:**
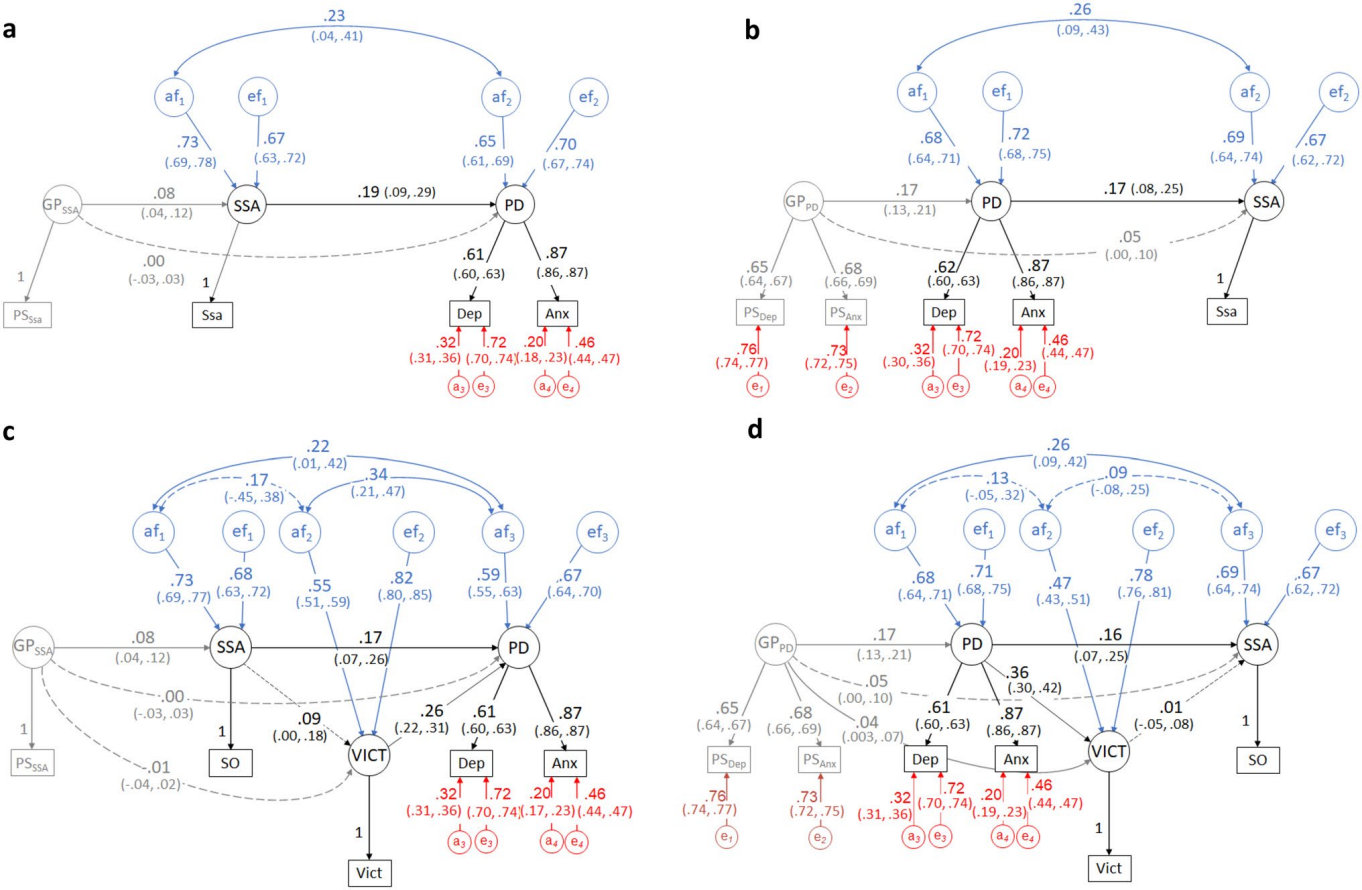
Mendelian Randomization-Direction of causation (MRDoC) models testing causal associations between same-sex attraction and psychological distress without and with victimization as a mediator including standardized path coefficients and 95% confidence intervals. *Note*. **a** MRDoC model 1: Causal influences of Same-Sex Attraction (SSA) on Psychological Distress (PD) with Genetic Propensity for Same-Sex Attraction (GP_SSA_) as instrument; **b** MRDoC model 2: Causal influences from PD towards SSA with Genetic Propensity for Psychological Distress (GP_PD_) as instrument; **c** MRDoC model 3: MRDoC Model 3: Model 1 with Victimization (VICT) included as a mediator; **d** MRDoC Model 4: Model 2 with Victimization included as a mediator. PS_Ssa_, PS_Dep_, PS_Anx_, Ssa, Dep and Anx = observed variables and indicators for the latent factors: Polygenic Scores for same-sex attraction, and for depressive and anxiety symptoms; Same-sex attraction, and Depressive and Anxiety symptoms respectively; af_1–2_, ef_1–2_ = additive genetic and individual-specific environmental influences on SSA and PD; _a3–4_ and e_3–4_ = residual additive genetic and individual-specific environmental influences on Depressive and Anxiety symptoms. The following constraints were specified for identification: factor loadings for single indicator factors (GP_SSA_ and SSA) were fixed to 1 and the corresponding residual variances were fixed to 0; for PD, the unstandardized factor loading on Dep was fixed to 1 while a_3_ and a_4_ and e_3_ and e_4_ were constrained to be equal respectively. Solid lines indicate significant paths while broken lines indicate non-significant paths

The phenotypic correlation between same-sex attraction and psychological distress was resolved into bidirectional causal effects in two separate models. MRDoC Model 1 (Fig. 3a) depicts causal influences of same-sex attraction on psychological distress (standardized path coefficient = 0.19, 95% CIs: 0.09, 0.29) while MRDoC Model 2 (Fig. 3b) depicts reverse causality between psychological distress and same-sex attraction (standardized path coefficient = 0.17, 95% CIs: 0.08, 0.25). Pleiotropic effects of same-sex attraction and psychological distress instruments (GP_SSA_ and GP_PD_ respectively) on psychological distress and same-sex attraction respectively in both models were not statistically significant. Considering that the coefficient of the pleiotropic effect of GPSSA on psychological distress was 0 (Fig. 3a), we tested the impact of rE (correlation between individual-specific environmental influences) on the causal path coefficient. For this, we constrained the pleiotropic path coefficient to 0 and freely estimated rE. The causal and correlation path coefficients in this exploratory model (β = 0.18 and rA = 0.24 respectively; 95% CIs: 0.16, 0.22 and −0.29, 0.61; and rE = 0) were comparable to those of the main model. These suggest that the causal path estimate was not confounded by unmeasured rE and that statistical power to estimate rA is reduced when rE is estimated.

#### Sex differences

The heterogeneity phenotypic factor correlation model fit the data better than the corresponding homogeneity model (χ^2^[52] = 877.60, *p* < 0.001). However, inspection of the correlation coefficients indicated that this difference likely reflected multiple small differences as all the confidence intervals of the parameter estimates overlapped. The homogeneity MRDoC models for MRDoC Models 1 and 2 (causal paths constrained to be equal in both sexes) were not significantly worse than the corresponding heterogeneity models (χ^2^[1] = 1.79, *p* = 0.18 and χ^2^[1] = 1.70, *p* = 0.19 respectively). This indicates that there were no significant sex differences in the causal effects. However, the large causal path estimates in males raises the possibility that the smaller absolute number of male participants may have reduced the power to estimate causal effects among them. Hence, we interpret this finding with caution.

#### Moderation by early-life adversity and childhood gender nonconformity

Although the causal effects of same-sex attraction on psychological distress increased with early-life adversity and childhood gender nonconformity (β = 0.02 and 0.03, 95% CIs: −0.02, 0.06 and −0.06, 0.11 respectively; Supplementary Table S3), these effects were not statistically significant. In contrast, the reverse causal path was significantly moderated by childhood gender nonconformity (β = −0.09, 95% CIs: −0.13, −0.04) but not early-life adversity (β = 0.05, 95% CIs: −0.01, 0.12). Specifically, reverse casual influences between psychological distress and same-sex attraction diminished as childhood gender nonconformity increased.

### Exploratory analyses

#### Mediation of causal paths by victimization

Regarding the mediated causal effect of same-sex attraction on psychological distress (MRDoC Model 3, Fig. 3c), the standardized coefficient of the first mediation path (Same-sex attraction → Victimization) was 0.09 but this was not statistically significant (95% CI: 0.00, 0.18) while the second mediation path (Victimization → Psychological distress) was significant (standardized path coefficient = 0.26, 95% CIs: 0.22, 0.31). We note the similar magnitude of the coefficients of the first mediation path and the instrumental path (0.08, 95% CIs: 0.04, 0.12) in this model. The statistical significance of the latter, in contrast to the former, suggests that the present study is underpowered to detect a true mediated causal effect. This may, in turn, be a consequence of specifying same-sex attraction as a liability threshold variable (Neale et al., 1994). We therefore focus on the magnitude of effect rather than statistical significance. The proportion of causal effect mediated in this model was 12.5%

In contrast, in the mediation model for the reverse causal path (MRDoC Model 4, Fig. 3d) the first mediation path (Psychological distress → Victimization) was statistically significant (standardized path coefficient = 0.36, 95% CIs: 0.30, 0.42) while the magnitude of the second mediation path (Victimization → Same-sex attraction) was very close to 0 (standardized path coefficient = 0.01, 95% CIs: −0.05, 0.08). The indirect effect was thus 0%, while the direct effect remained statistically significant.

## Discussion

The present study demonstrated bidirectional causal relationships between same-sex attraction (indexing sexuality-related stress) and psychological distress, consistent with the finding from a previous study (Oginni et al., 2022a). These effects did not appear to vary significantly by sex. Reciprocal causation between psychological distress and same-sex attraction was weaker at higher levels of childhood gender nonconformity. Finally, our findings suggest that victimization may partially mediate the causal effect of same-sex attraction on psychological distress but not the reverse causal path.

The causal influence of same-sex attraction on psychological distress demonstrated in the present study is consistent with prior evidence indicating that the phenotypic associations between same-sex attraction and adverse health outcomes are not confounded by correlated genetic and environmental influences (Oginni et al., 2020; Oginni et al., 2022c cf. Bailey, 2020). This contrasts with previous evidence of genetic and environmental correlations between same-sex attraction and depression (Frisell et al., 2010; Ganna et al., 2019; Zietsch et al., 2012). Specifically, our analyses demonstrated very small independent associations between same-sex attraction polygenic scores and psychological distress, and vice versa. Thus, the previously described genetic correlations may reflect vertical pleiotropy (i.e., genetic influences transmitted through phenotypic causal paths) rather horizontal pleiotropy (a common set of genetic factors independently influencing multiple outcomes; Oginni, et al., 2020, 2022c; Paaby & Rockman, 2013). The causal effect of same-sex attraction on psychological distress is also consistent with the minority stress theory in which stress associated with sexual minority status causes higher mental health adversities through intermediary affective, cognitive and behavioral processes (Feinstein, 2020; Hatzenbuehler, 2009; Meyer, 2003). This explanation is further validated by our finding that victimization explained 12.5% of the causal effect of same-sex attraction on psychological distress. The lack of statistical significance for this mediation effect is likely due to low power from specifying same-sex attraction as a liability threshold variable (Neale et al., 1994) and we interpret this effect with caution. The large direct (unmediated) effect suggests the role of other mediators (such as other minority stress factors) or confounders in this causal path.

The reverse causal effect of psychological distress on same-sex attraction (indexing sexuality-related stress) is consistent with a previous finding (Oginni et al., 2022a). However, although victimization was significantly causally influenced by psychological distress which was consistent with existing literature (Hart et al., 2012; Maniglio, 2009); it did not mediate this reverse causal path. Taken together, the separate significant causal associations between psychological distress and victimization are consistent with a feedback loop whereby sexuality-related victimization results in psychological distress which in turn increases the likelihood of further victimization. The large unmediated reverse causal effect (psychological distress → same-sex attraction) suggests that the mechanisms whereby psychological distress reinforces sexuality-related stress are independent of victimization. Alternatively, this unmediated effect may reflect phenotypic confounding by a variable that is associated with psychological distress, victimization and same-sex attraction. One such variable is childhood gender nonconformity which is associated with later same-sex attraction (Li et al., 2017; Xu et al., 2021), victimization and psychological distress (D’Augelli et al., 2006; Jones et al., 2017). However, when we controlled for childhood gender nonconformity as a covariate in mediation analyses, the causal and mediation path coefficients were largely unchanged. Another alternative possibility is that the unmediated reverse causal path reflects other minority stressors that are more proximal to same-sex attraction such as internalized and perceived stigma (Meyer, 2003) but this needs to be specifically tested.

### Sex differences

Similarly, although previous studies suggested sex differences in the mental health disparities among sexual minority compared to heterosexual individuals (King et al., 2008); the present study found that the unmediated bidirectional causal paths were comparable in men and women. Our finding may suggest that the causal mechanisms of disparities in psychological distress among sexual minorities do not differ by sex. The sex differences found in earlier observational studies may reflect differences in the experience of minority stressors in sexual minority men compared to women. For example, in the present study, the median victimization score was higher among male compared to female participants. However, considering the large confidence intervals for the causal path coefficient among men, we recognize the possibility that our sample was too small to detect significant sex differences.

### Moderation by early-life adversities and childhood gender nonconformity

We further demonstrated that the reciprocal causal path from psychological distress towards same-sex attraction weakened as childhood gender nonconformity increased. This suggests a protective effect whereby the feedback link of psychological distress on putative minority stress processes is attenuated among those with higher childhood gender nonconformity. For example, a recent meta-analysis found that gender nonconformity is significantly associated with lower internalized homonegativity (Thoma et al., 2021). Similarly, another recent study found that retrospectively-assessed childhood gender nonconformity attenuated individual-specific influences on the association between non-heterosexuality and anxiety symptoms (Oginni et al., 2022b). Although the causal effects of same-sex attraction on psychological distress appeared stronger with higher early-life adversities and childhood gender nonconformity, these effects were not statistically significant.

## Strengths and limitations

The combination of genomic data with cross-sectional twin data enabled us to test bidirectional causal associations. The assessment of victimization in both same-sex attracted and heterosexual participants enabled the estimation of how much it contributed to cross-group differences in adverse health outcomes. The use of prospectively-assessed childhood gender nonconformity also helped minimize any effects of recall bias in this trait. However, despite these strengths, our findings need to be interpreted in the light of the following limitations.

The specification of same-sex attraction as a liability threshold variable is consistent with common practice for categorical variables (Neale et al., 1994). However, this specification reduced our power to investigate mediation of the causal effects and sex differences in these. We also tested for bidirectional causal effects in separate models as has been previously done (Oginni et al., 2022a) but these would have been better tested simultaneously in a single model. However, such a model would require assumptions such as the absence of pleiotropy that may bias causal path estimates (Castro-de-Araujo et al., 2022). Bidirectional causal effects may be tested simultaneously using other designs such as the direction of causation twin model (under certain conditions, Heath et al., 1993) and the longitudinal cross-lagged model (Burt et al., 2005).

Even though moderation of the causal paths by early-life adversity was not statistically significant, we note that intrapersonal factors such as significant psychological distress and personality traits like neuroticism may introduce recall bias, i.e., increase the likelihood of reporting or interpreting past events as traumatic (Larsen, 1992). Hence, caution should be taken in future studies replicating our analyses using larger sample sizes. Such studies may specify multiple measures of recalled adversity as indicators of a latent adversity measure (Heath et al., 1993; Schoemann & Jorgensen, 2021) or estimate individual-specific environmental correlation alongside the causal path coefficient if the model will be identified.

Although victimization and other stressful experiences are typically higher among sexual minorities, and are attributable to their sexual minority status or related traits like childhood gender nonconformity (King et al., 2003; Roberts et al., 2013), victimization in the present study was not specifically associated with sexual minority status. Thus, victimization in the present study may reflect other factors than same-sex attraction. Similarly, while we use same-sex attraction as an index of sexuality-related distress, future studies should incorporate specific indices in their analytic models.

## Future directions

Future research can improve on the present study by incorporating other minority stressors such as internalized sexuality-related stigma (Meyer, 2003), and related mechanisms such as rumination (Timmins et al., 2020) and rejection sensitivity (Feinstein, 2020) as mediators of the demonstrated causal effects. These can potentially explain some of the unmediated direct effects in both causal paths. However, larger samples would be required for such analyses, and the investigation of sex differences in these mediation effects. Power may also be increased by assessing same-sex sexuality as a continuous measure.

As recommended in the description of the MRDoC model (Minică et al., 2018), we suggest the use of alternative research designs to provide external validation for the causal effects demonstrated in the present study (e.g., de Vries et al., 2021). Such designs may include biometric or phenotypic longitudinal cross-lagged models (Burt et al., 2005; Hamaker et al., 2015) which can simultaneously test bidirectional causal effects and be extended to test for causal mediation (Kline, 2015).

## Conclusions

We demonstrated bidirectional causal effects between same-sex attraction (indexing sexuality-related stress) and psychological distress with no significant sex differences in these effects. Moderation analyses suggested a protective effect of childhood gender nonconformity on the effect of psychological distress on minority stress. Further mediation analyses suggested a feedback loop whereby minority stress increases the likelihood of psychological distress among sexual minorities which in turn reinforces minority stress.

The causal effect of same-sex attraction on psychological distress and mediation by victimization in the present study highlight the importance of systemic interventions targeted at reducing sexuality-based discrimination to improve the overall wellbeing of sexual minority individuals. The feedback loop between psychological distress and victimization in the context of sexual minority status further suggests the importance of addressing sexual minority stress during individual interventions for psychological distress among sexual minorities. The protective mechanisms of childhood gender nonconformity in this feedback process need to be specifically investigated.

## Supplementary Information

Below is the link to the electronic supplementary material.

Supplementary file1 (DOCX 85 KB)

## Data Availability

The data are available on request from the TEDS team at: https://www.teds.ac.uk/researchers/teds-data-access-policy.

## Footnotes

1 Although these polygenic scores were based on a genome-wide association study for same-sex behavior, we describe them as ‘polygenic scores for same-sex attraction’ for ease of presentation.

## Code availability

The code for analysis is freely available at: https://github.com/Kaoginni/MRDoC-mediation-and-moderation/blob/main/MRDoC%20-%20mediation,%20moderation,%20sex%20differences.R
