# Supplementary material for: Bidirectional Causal Associations Between Same-Sex Attraction and Psychological Distress: Testing Moderation and Mediation Effects
Source: Behav Genet. 2022 Dec 15;53(2):118–31. doi: 10.1007/s10519-022-10130-x (PMC9922221; doi:10.1007/s10519-022-10130-x)
Supplement: Supplementary file 1 — Supplementary file1 (DOCX 85 KB) [file 10519_2022_10130_MOESM1_ESM.docx]

Supplementary material for

Bidirectional causal associations between same-sex attraction and psychological distress: Testing moderation and mediation effects

Method Page 2

Results Page 3

Figure S1: Mediated Mendelian Randomisation-Direction of Causation model

(MRDoC Model 3) with Victimization (VICT) mediating the causal effect

of Same-sex attraction (SSA) on Psychological distress (PD) Page 3

Figure S2: Moderated Mendelian Randomisation-Direction

of Causation (MRDoC) model Page 4

Table S1: Complete and incomplete twin pairs by sex and zygosity per variable Page 5

Table S2: Phenotypic correlations between study variables and

95% confidence intervals Page 6

Table S3: Standardized additive genetic, shared and individual-specific environmental influences on the variances and covariances between the latent factors Page 8

Table S4a: Phenotypic correlation between moderators and other study

variables in males and females Page 9

Table S4b: Sex differences and moderation of the causal paths between same-sex

attraction and psychological distress and 95% confidence intervals Page 10

Table S5a: Phenotypic correlations between latent factors standardized

factor loadings and residual variance paths in male participants Page 11

Table S5b: Phenotypic correlations between latent factors standardized

factor loadings and residual variance paths in female participants Page 12

**Method**

**Calculating polygenic scores**

Genotype data were collected at ages 12- and 16-years using cheek swab and saliva samples respectively. These were analyzed using AffymetrixGeneChip 6.0 SNP arrays (3665 individuals) and Illumina HumanOmniExpressExome-8v1.2 arrays (8122 individuals). Only one twin per monozygotic pair was genotyped as monozygotic twins are held to be genetically identical. The genotypes from both platforms were imputed using the Haplotype Reference Consortium (release 1.1) through the Sanger Imputation Service (McCarthy et al., 2016). In total, 10,346 samples, and 635,269 Single Nucleotide Polymorphisms – SNPs (AffymetrixGeneChip) and 559,772 SNPs (Illumina HumanOmniExpressExome) passed quality control (Selzam et al., 2019; <https://teds.ac.uk/datadictionary/studies/dna.htm>).

Polygenic scores were calculated for same-sex attraction, depressive and anxiety symptoms as the weighted sum of the number of genome-wide trait-associated alleles weighted by effect sizes using the Bayesian-based LDpred (Vilhjálmsson et al., 2015). In LDpred, a fraction of the SNPs associated with a trait are selected as priors and used as weights while the other SNPs are weighted approximately 0. In addition, linkage disequilibrium is accounted for using a reference panel. Polygenic scores are iteratively calculated using varying fractions of SNPs to identify the proportion of genetic variants that predicts the greatest trait variance. Effect sizes were derived from genome-wide association studies for same-sex attraction (Ganna et al., 2019), and depressive (Howard et al., 2019) and anxiety symptoms (Purves et al., 2020). The respective fraction of SNPs included to determine the final polygenic scores for each of these variables were 0.5, 1 and 0.3 respectively.

**Results**

Figure S1:

Mediated Mendelian Randomisation-Direction of Causation model (MRDoC Model 3) with Victimization (VICT) mediating the causal effect of Same-sex attraction (SSA) on Psychological distress (PD)


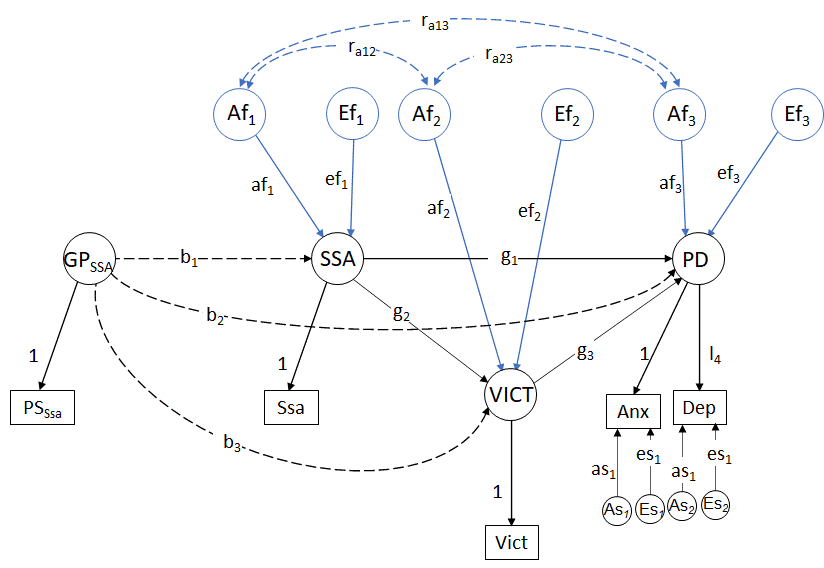


*Note*. Genetic Propensity for SSA (GP_SSA_, indicated by polygenic score for Same-Sex Attraction – PS_Ssa_) specified as instrument for SSA; b_1-3_ indicate instrumental path to SSA and pleiotropic paths to PD and VICT respectively; g_1_ = causal paths, g_2-3_ = causal mediation paths. Af_1-3_, Cf_1-3_ and Ef_1-3_ = Additive genetic and shared and individual-specific environmental influences on SSA, VICT and PD respectively; af_1-3_, cf_1-3_ and ef_1-3_ = their path coefficients; ra_12_, ra_13_ and ra_23_, and rc_12_, rc_13_ and rc_23_ = correlation coefficients between Af_1-3_ and Cf_1-3_ respectively; As_1_, Cs_1_ and Es_1_ = Residual additive genetic and shared and individual-specific environmental influences on Depressive (Dep) and Anxiety symptoms (Anx) which are constrained to be equal for identification; and as_1_, cs_1_ and es_1_ = their path coefficients. In MRDoC Model 4, PD and SSA were specified as the predictor and outcome respectively and Genetic Propensity for Psychological Distress (GP_PD_), as the instrument. C parameters (Cf_1_, Cf_2_, Cf_3_, rc_12_, rc_13_, rc_23_ and Cs_1_) are not depicted for clarity and because these are not included in the full model. Correlation paths between Ef_1-3_ (i.e., re_12_, re_13_ and re_23_) are fixed to 0 to identify the causal mediation paths.

Figure S2:

Moderated Mendelian Randomisation-Direction of Causation (MRDoC) model


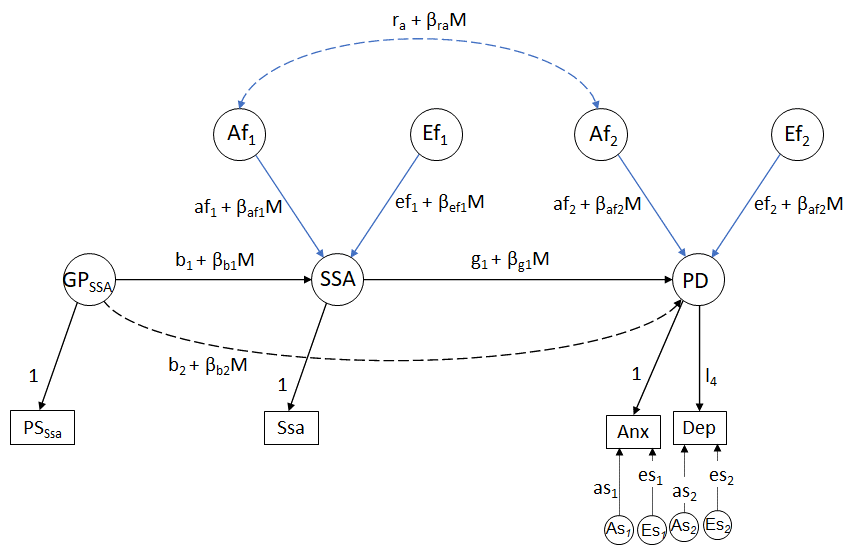


*Note*. MRDoC model with moderation terms (β) specified for the instrumental, pleiotropic and causal paths (b_1_, b_2_ and g_1_ respectively), the variance component path coefficients (af_1_, af_2_, ef_1_ and ef_2_) and the covariance path between the additive genetic influences (ra). Af_1_, Af_2_, Ef_1_, Ef_2_ = Additive genetic and individual-specific environmental influences on Same-Sex Attraction (SSA) and Psychological Distress (PD); af_1_, af_2_, ef_1_ and ef_2_ = their path coefficients respectively. Genetic propensity for SSA (GP_SSA_, indicated by Polygenic Score for Same-Sex Attraction – PS_Ssa_) specified as instrument for SSA; As_1-2_, Cs_1-2_ and Es_1-2_ = Residual additive genetic and shared and individual-specific environmental influences on Depressive (Dep) and Anxiety symptoms (Anx) respectively; and as_1-2_, cs_1-2_ and es_1-2_ = their path coefficients, l_4_ = factor loading of PD on Dep.

Table S1:

Complete and incomplete twin pairs by sex and zygosity per variable

| Variable | MZM  Complete/  Incomplete | DZM  Complete/  Incomplete | MZF  Complete/  Incomplete | DZF  Complete/  Incomplete | DZO  Complete/  Incomplete | Complete MZ/DZ | Total |
| --- | --- | --- | --- | --- | --- | --- | --- |
| Same-sex attraction | 479/182 | 389/242 | 964/252 | 772/314 | 1072/683 | 1443/2233 | 9025 |
| Depressive symptoms | 479/185 | 403/232 | 975/242 | 785/305 | 1087/682 | 1454/2275 | 9104 |
| Anxiety symptoms | 423/176 | 321/261 | 896/248 | 731/294 | 900/705 | 1319/1952 | 8226 |
| Victimisation | 410/150 | 316/222 | 868/252 | 699/303 | 864/674 | 1278/1879 | 7915 |
| Polygenic scores* | -/968 | 662/212 | -/1131 | 731/175 | 1259/382 | -/2652 | 8172 |
| CGN (4 years)† | 1195/34 | 1252/41 | 1378/41 | 1267/27 | 2442/88 | 2573/4961 | 15355 |
| ELA | 429/187 | 321/270 | 909/249 | 732/297 | 922/709 | 1338/1975 | 8338 |

*Note*. *For monozygotic twins, only one individual per pair was genotyped as they are held to be genetically identical.

MZM and MZF = Monozygotic same-sex male and female twin pairs respectively, DZM, DZF, and DZO = Dizygotic same-sex male, female and opposite-sex twin pairs respectively.

CGN = Childhood gender nonconformity, ELA = Early-life adversities

†The sample size is relatively larger because the assessment was at an earlier time point – 4 years, with relatively greater participation.

Table S2:

Phenotypic correlations between study variables and 95% confidence intervals

| Variables | SSA  (1) | Dep  (2) | Anx  (3) | Vict  (4) | PS_SSA_  (5) | PS_Dep_  (6) | PS_Anx_  (7) |
| --- | --- | --- | --- | --- | --- | --- | --- |
| Within-person correlations |  |  |  |  |  |  |  |
| (1) | 1 |  |  |  |  |  |  |
| (2) | .22  (.19, .25) | 1 |  |  |  |  |  |
| (3) | .24  (.21, .27) | .53  (.52, .55) | 1 |  |  |  |  |
| (4) | .16  (.13, .19) | .28  (.26, .30) | .35  (.33, .37) | 1 |  |  |  |
| (5) | .08  (.04, .12) | .01  (-.02, .04) | .01  (-.02, .04) | -.01  (-.04, .02) | 1 |  |  |
| (6) | .07  (.03, .11) | .12  (.09, .14) | .11  (.08, .14) | .09  (.07, .12) | .12  (.09, .14) | 1 |  |
| (7) | .04  (.00, .08) | .07  (.04, .09) | .07  (.04, .10) | .04  (.01, .07) | .10  (.07, .12) | .43  (.42, .45) | 1 |
| Between twin correlations |  |  |  |  |  |  |  |
| Monozygotic |  |  |  |  |  |  |  |
| (1) | .56  (.49, .63) |  |  |  |  |  |  |
| (2) | .15  (.11, .19) | .33  (.29, .37) |  |  |  |  |  |
| (3) | .16  (.11, .20) | .30  (.26, .33) | .40  (.35, .44) |  |  |  |  |
| (4) | .12  (.07, .17) | .17  (.13, .20) | .18  (.15, .22) | .34  (.29, .38) |  |  |  |
| (5) | .08^a^  (.04, .12) | .01^a^  (-.02, .04) | .01^a^  (-.02, .04) | -.01^a^  (-.04, .02) | 1^a^ |  |  |
| (6) | .07^a^  (.03, .11) | .12^a^  (.09, .14) | .11^a^  (.08, .14) | .09^a^  (.07, .12) | .12^a^  (.09, .14) | 1^a^ |  |
| (7) | .04^a^  (.00, .08) | .07^a^  (.04, .09) | .07^a^  (.04, .10) | .04^a^  (.01, .07) | .10^a^  (.07, .12) | .43^a^  (.42, .45) | 1^a^ |
| Dizygotic |  |  |  |  |  |  |  |
| (1) | .22  (.15, .30) |  |  |  |  |  |  |
| (2) | .10  (.06, .14) | .18  (.14, .22) |  |  |  |  |  |
| (3) | .11  (.07, .15) | .14  (.10, .17) | .16  (.11, .20) |  |  |  |  |
| (4) | .05  (.01, .09) | .09  (.06, .12) | .09  (.06, .12) | .14  (.10, .18) |  |  |  |
| (5) | .02  (-.03, .06) | -.01  (-.05, .02) | .01  (-.03, .04) | -.02  (-.05, .01) | .50  (.47, .53) |  |  |
| (6) | .00  (-.05, .04) | .05  (.02, .09) | .05  (.01, .08) | .05  (.02, .08) | .06  (.03, .08) | .52  (.50, .55) |  |
| (7) | .05  (.01, .10) | .04  (.01, .07) | .06  (.03, .10) | .02  (-.01, .05) | .05  (.02, .08) | .22  (.19, .24) | .48  (.45, .50) |

*Note*. SSA=Same-Sex Attraction; Dep=Depressive symptoms; Anx=Anxiety symptoms; Vict=Victimization; PS_SSA_, PS_Dep_ and PS_Anx_=Polygenic Scores for Same-Sex Attraction and Depressive and Anxiety symptoms respectively; ^a^Correlation coefficients constrained to be equal to within-person coefficients because only one individual of each monozygotic twin pair was genotyped and the monozygotic twin pairs are held to be approximately 100% genetically identical.

Table S3:

Standardized additive genetic, shared and individual-specific environmental influences on the variances and covariances between the latent factors

|  | Same-sex attraction (1) | Psychological Distress (2) | Victimization (3) |
| --- | --- | --- | --- |
| h^2^ |  |  |  |
| 1. | .54 (.40, .60) |  |  |
| 2. | .71 (.40, .86) | .54 (.43, .60) |  |
| 3. | .74 (.32, .90) | .55 (.39, .65) | .32 (.23, .36) |
| c^2^ |  |  |  |
| 1. | .00 (.00, .11) |  |  |
| 2. | .00 (-.07, .23) | .00 (.00, .09) |  |
| 3. | .00 (-.25, .12) | .00 (-.05, .12) | .00 (.00, .06) |
| e^2^ |  |  |  |
| 1. | .46 (.40, .53) |  |  |
| 2. | .29 (.15, .43) | .46 (.40, .51) |  |
| 3. | .26 (.10, .52) | .45 (.37, .53) | .68 (.64, .72) |
|  |  |  |  |
| Variables | As | Cs | Es |
| Same-sex attraction | - | - | - |
| Depressive symptoms | .07 (.00, .12) | .02 (.00, .09) | .47 (.43, .51) |
| Anxiety symptoms | .04 (.00, .07) | .00 (.00, .03) | .32 (.28, .36) |
| Victimization | - | - | - |

*Note*. h^2^, c^2^ and e^2^ = standardized additive genetic, and shared and individual-specific environmental influences on factor variances (the diagonals) and covariances (the off-diagonals).

Table S4a:

Phenotypic factor correlations between moderators (early-life adversity and childhood gender nonconformity) and other study variables (same-sex attraction, psychological distress and victimization) in males (below the diagonal) and females (above the diagonal).

| Latent factors | Childhood gender nonconformity  (1) | Early-life adversity  (2) | Same-sex attraction  (3) | Psychological distress  (4) | Victimization  (5) |
| --- | --- | --- | --- | --- | --- |
| (1) | 1 | .08  (.05, .12) | .20  (.16, .24) | .02  (-.02, .06) | .05  (.02, .08) |
| (2) | .00  (-.04, .04) | 1 | .21  (.18, .25) | .34  (.31, .37) | .31  (.28, .33) |
| (3) | .30  (.25, .36) | .15  (.10, .20) | 1 | .32  (.28, .36) | .19  (.15, .22) |
| (4) | .07  (.03, .12) | .31  (.27, .35) | .29  (.23, .34) | 1 | .45  (.42, .48) |
| (5) | .06  (.02, .10) | .29  (.26, .33) | .13  (.08, .19) | .42  (.38, .46) | 1 |

Table S4b:

Sex differences and moderation of the causal paths between same-sex attraction and psychological distress and 95% confidence intervals

|  | Sex | | Moderator | |
| --- | --- | --- | --- | --- |
| Relationship | Male  β (95% CI) | Female  β (95% CI) | Early-life adversity  β (95% CI) | Childhood gender nonconformity  β (95% CI) |
| SSA→PD | 0.33 (0.15, 0.52) | 0.15 (0.04, 0.26) | 0.02 (-0.02, 0.06) | 0.03 (-0.06, 0.11) |
| PD→SSA | 0.23 (0.08, 0.39) | 0.13 (0.02, 0.24) | 0.05 (-0.01, 0.12) | -0.09 (-0.13, -0.04) |

*Note*. β = Standardized coefficient, SSA = Same-sex attraction, PD = Psychological Distress;

Table S5a:

Phenotypic correlations between latent factors standardized factor loadings and residual variance paths in male participants

|  | Same-sex attraction (1) | Psychological distress (2) | Victimization (3) | GP_SSA_  (4) | GP_PD_  (5) |
| --- | --- | --- | --- | --- | --- |
| Within-person |  |  |  |  |  |
| 1. | 1.00 |  |  |  |  |
| 2. | .28 (.22, .34) | 1.00 |  |  |  |
| 3. | .13 (.08, .19) | .42 (.38, .46) | 1.00 |  |  |
| 4. | .10 (.03, .17) | .01 (-.04, .06) | .01 (-.04, .06) | 1.00 |  |
| 5. | .06 (-.03, .14) | .18 (.12, .25) | .12 (.06, .18) | .16 (.12, .20) | 1.00 |
| Cross-twin |  |  |  |  |  |
| Monozygotic |  |  |  |  |  |
| 1. | .55 (.41, .66) |  |  |  |  |
| 2. | .10 (.00, .20) | .50 (.39, .59) |  |  |  |
| 3. | .08 (-.01, 0.17) | .18 (.10, .25) | .26 (.17, .35) |  |  |
| 4. | .10 (.03, .17)^a^ | .01 (-.04, .06)^a^ | .01 (-.04, .06)^a^ | 1.00 |  |
| 5. | .06 (-.03, .14)^a^ | .18 (.12, .25)^a^ | .12 (.06, .18)^a^ | .16 (.12, .20)^a^ | 1.00 |
| Dizygotic |  |  |  |  |  |
| 1. | .17 (-.06, .40) |  |  |  |  |
| 2. | .04 (-.09, .17) | .24 (.12, .36) |  |  |  |
| 3. | -.08 (-.19, .04) | .08 (-.01, .16) | .14 (.03, .24) |  |  |
| 4. | .08 (-.03, .18) | .03 (-.05, .10) | .03 (-.04, .10) | .50 |  |
| 5. | .00 (-.16, .16) | .05 (-.06, .16) | .06 (-.04, .17) | .05 (-.01, .11) | .50 |
|  |  |  |  |  |  |
| Variables | Standardized factor loadings | Standardized error variance path |  |  |  |
| Same-sex attraction | 1.00 | - |  |  |  |
| Dep symptoms | .64 (.62, .67) | .76 (.74, .79) |  |  |  |
| Anx symptoms | .85 (.84, .86) | .53 (.51, .55) |  |  |  |
| Victimization | 1.00 | - |  |  |  |
| PS_SSA_ | 1.00 | - |  |  |  |
| PS_Dep_ | .67 (.65, .69) | .74 (.72, .76) |  |  |  |
| PS_Anx_ | .64 (.62, .66) | .77 (.75, .79) |  |  |  |

*Note*. GP_SSA_, GP_PD_ = Genetic Propensities for Same-Sex Attraction and Psychological Distress respectively; Dep and Anx symptoms = Depressive and Anxiety symptoms respectively; PS_SSA_, PS_Dep_ and PS_Anx_ = Polygenic scores for Same-Sex Attraction, Depressive symptoms and Anxiety symptoms. The factor loadings for factors with single indicator variables (Same-Sex Attraction, Victimization and GP_SSA_) were fixed to 1 while the residual variances were fixed to 0 for identification. ^a^Correlation coefficients constrained to be equal to within-person coefficients because only one individual of each monozygotic twin pair was genotyped and the monozygotic twin pairs are held to be approximately 100% genetically identical.

Table S5b:

Phenotypic correlations between latent factors standardized factor loadings and residual variance paths in female participants

|  | Same-sex attraction (1) | Psychological distress (2) | Victimization  (3) | GP_SSA_  (4) | GP_PD_  (5) |
| --- | --- | --- | --- | --- | --- |
| Within-person |  |  |  |  |  |
| 1. | 1.00 |  |  |  |  |
| 2. | .32 (.28, .36) | 1.00 |  |  |  |
| 3. | .19 (.15, .23) | .45 (.42, .48) | 1.00 |  |  |
| 4. | .07 (.02, .11) | .01 (-.03, .05) | -.02 (-.05, .02) | 1.00 |  |
| 5. | .10 (.04, .16) | .17 (.12, .22) | .09 (.04, .14) | .16 (.12, .20) | 1.00 |
| Cross-twin |  |  |  |  |  |
| Monozygotic |  |  |  |  |  |
| 1. | .56 (.47, .64) |  |  |  |  |
| 2. | .24 (.18, .30) | .57 (.51, .63) |  |  |  |
| 3. | .16 (.10, .21) | .28 (.23, .32) | .38 (.33, .44) |  |  |
| 4. | .07 (.02, .11)^a^ | .01 (-.03, .05)^a^ | -.02 (-.05, .02)^a^ | 1.00 |  |
| 5. | .10 (.04, .16)^a^ | .17 (.12, .22)^a^ | .09 (.04, .14)^a^ | .16 (.12, .20)^a^ | 1.00 |
| Dizygotic |  |  |  |  |  |
| 1. | .27 (.16, .38) |  |  |  |  |
| 2. | .18 (.11, .25) | .28 (.19, .36) |  |  |  |
| 3. | .08 (.02, .15) | .14 (.08, .20) | .20 (.13, .27) |  |  |
| 4. | .02 (-.05, .09) | .01 (-.05, .07) | -.06 (-.12, .00) | .50 |  |
| 5. | .00 (-.10, .10) | .11 (.02, .19) | -.03 (-.11, .05) | .07 (.02, .13) | .50 |
|  |  |  |  |  |  |
| Variables | Standardized factor loadings | Standardized error variance path |  |  |  |
| Same-sex attraction | 1.00 | .00 |  |  |  |
| Dep symptoms | .62 (.60, .63) | .79 (.77, .80) |  |  |  |
| Anx symptoms | .87 (.86, .88) | .49 (.48, .51) |  |  |  |
| Victimization | 1.00 | .00 |  |  |  |
| PS_SSA_ | 1.00 | .00 |  |  |  |
| PS_Dep_ | .69 (.67, .71) | .73 (.71, .75) |  |  |  |
| PS_Anx_ | .66 (.64, 68) | .75 (.73, .77) |  |  |  |

*Note*. GP_SSA_, GP_PD_ = Genetic Propensities for Same-Sex Attraction and Psychological Distress respectively; Dep and Anx symptoms = Depressive and anxiety symptoms respectively; PS_SSA_, PS_Dep_ and PS_Anx_ = Polygenic Scores for Same-Sex Attraction, Depressive symptoms and Anxiety symptoms. The factor loadings for factors with single indicator variables (Same-Sex Attraction, Victimization and GP_SSA_) were fixed to 1 while the residual variances were fixed to 0 for identification. ^a^Correlation coefficients constrained to be equal to within-person coefficients because only one individual of each monozygotic twin pair was genotyped and the monozygotic twin pairs are held to be approximately 100% genetically identical.

**References**

Ganna, A., Verweij, K. J., Nivard, M. G., Maier, R., Wedow, R., Busch, A. S., Abdellaoui, A., Guo, S., Sathirapongsasuti, J. F., & 16, a. R. T. (2019). Large-scale GWAS reveals insights into the genetic architecture of same-sex sexual behavior. *Science*, *365*(6456), eaat7693. <https://doi.org/10.1126/science.aat7693>

Howard, D. M., Adams, M. J., Clarke, T.-K., Hafferty, J. D., Gibson, J., Shirali, M., Coleman, J. R., Hagenaars, S. P., Ward, J., & Wigmore, E. M. (2019). Genome-wide meta-analysis of depression identifies 102 independent variants and highlights the importance of the prefrontal brain regions. *Nature Neuroscience*, *22*(3), 343-352. <https://doi.org/10.1038/s41593-018-0326-7>

McCarthy, S., Das, S., Kretzschmar, W., Delaneau, O., Wood, A. R., Teumer, A., Kang, H. M., Fuchsberger, C., Danecek, P., & Sharp, K. (2016). A reference panel of 64,976 haplotypes for genotype imputation. *Nature Genetics*, *48*(10), 1279. <https://doi.org/10.1038/ng.3643>

Purves, K. L., Coleman, J. R., Meier, S. M., Rayner, C., Davis, K. A., Cheesman, R., Bækvad-Hansen, M., Børglum, A. D., Wan Cho, S., & Jürgen Deckert, J. (2020). A major role for common genetic variation in anxiety disorders. *Molecular Psychiatry*, *25*(12), 3292-3303. <https://doi.org/10.1038/s41380-019-0559-1>

Selzam, S., Ritchie, S. J., Pingault, J.-B., Reynolds, C. A., O’Reilly, P. F., & Plomin, R. (2019). Comparing within-and between-family polygenic score prediction. *The American Journal of Human Genetics*, *105*(2), 351-363. <https://doi.org/10.1016/j.ajhg.2019.06.006>

Vilhjálmsson, B. J., Yang, J., Finucane, H. K., Gusev, A., Lindström, S., Ripke, S., Genovese, G., Loh, P.-R., Bhatia, G., & Do, R. (2015). Modeling linkage disequilibrium increases accuracy of polygenic risk scores. *The American Journal of Human Genetics*, *97*(4), 576-592. <https://doi.org/10.1016/j.ajhg.2015.09.001>
